# Annotations

**Published:** 1897-06-12

**Authors:** 


### ANNOTATIONS.

### A Jubilee Eviction.

MUCH sympathy has been expressed for the unfortunate tenants along the route of the Royal Procession who have been threatened with eviction. In fact, an Act of Parliament has been passed to prevent such tyrannous proceedings. What, then, are we to think of the eviction of the patients at the Royal Eye Hospital, the closing of its out-patient department, the cessation of the work of the charity, in order that the institution may be turned into a grand stand, and that money may be earned by the letting of Jubilee seats? Truly the ways of charity are mysterious, and the ins and outs of hospital management are difficult to understand. Here we have a hospital, founded with great *éclat*, said to be doing a great and necessary work, and providing for a pressing want. Yet so little do the managers appear to be impressed by the touching appeals they have issued to the public, so little do they seem to appreciate the necessity of the work they have undertaken, that they have not hesitated to put a sudden stop to their works of charity for the sake of letting seats. We cannot believe that any committee which had a proper conception of its responsibilities, and of the obligations lying upon it to carry out the objects of the charity, would deal so lightly with the wants and requirements—we might almost say the right—of its patients, and it becomes a matter of some importance to inquire as to the inner working of this institution. House committees require to be constantly reminded that they are responsible to the subscribers and to the public for the good management of the hospitals they control; that they have no right to delegate their duties to any individual, however energetic and interested he may seem; and that when things go wrong they must bear the blame. If the Royal Eye Hospital is short of money let it appeal to the charitable public. It will not appeal in vain if it can show that it supplies a want. We cannot, however, but feel that a hospital which can close its doors for the sake of letting seats thereby throws doubts upon the necessity for its existence.

### The Workmen's Compensation Bill.

IN commenting upon this Bill on the occasion of its introduction we pointed out the difficulty that might arise in regard to the meaning which would be attached to the term "incapacity for work." We asked, what is to be done with those whose accidents, while incapacitating them from doing their old work, leaves them fairly able to get along in some other way? and we pointed out how strict the friendly societies had found it necessary to be in preventing such men from eking out their sick pay by doing odd work. In the Committee stage this difficulty has been got over by Mr. Chamberlain, on whose motion the following subsection was inserted:—"In fixing the amount of the weekly payment regard shall be had to the difference between the amount of the weekly earnings of the workman before the accident and the amount which he is able to earn after the accident." This would seem to meet the case in a very satisfactory manner. Sir A. Hickman proposed to add the following words:—"Provided that he obtains from the medical officer of health for the district in which he resides, or from the medical officer appointed by his employers for the purpose, weekly a certificate of such disablement," but as Sir M. W. Ridley declined to accept this the amendment was withdrawn, and very properly so. No doubt as a man goes on towards recovery, or as his earnings in his new position gradually approach towards what he was earning before, his allowance will occasionally require revision; but the imposition of the necessity of obtaining a medical certificate every week would have been a great hardship. So far as the profession is concerned, we feel strongly that nothing has tended so much to bring medical certificates into contempt as the frequency with which they are asked for, and we fear are given, as a merely formal preliminary to the drawing of the weekly allowance. We are very glad, therefore, that the proposal to demand weekly certificates was negatived.

### Deaths from Chloroform.

WE have again to record a long string of deaths from chloroform. Our last allusion to this subject was on April 24th, when we recorded five deaths from anæsthetics which had occurred in the first ten days of that month. We now continue the sad list. The *Manchester Courier* of April 19th reports an inquest concerning the death of a man, aged 54, which took place in the infirmary on April 13th, at the conclusion of an operation for cancer of the mouth, chloroform having been administered. The *Yorkshire Post* of April 20th reports the case of a blacksmith, aged 54, who died under chloroform at the Sunderland Infirmary on April 17th. Death took place before the operation (for opening an abscess) was commenced. The *Sussex Daily News*, April 19th, reports the death of a woman, aged 36, at the Tunbridge Wells Hospital on April 15th. In this case also the death took place before the operation was commenced, the patient becoming very pale, and all efforts to restore the action of the heart proving futile. The *Standard*, April 26th, reports the death of a gymnast, aged 16, at St. Thomas's Hospital while under chloroform. The operation had been performed, and the patient, who had had from three to four drachms of chloroform, was coming round, when the heart failed. The *Manchester Courier*, April 28th, reports the death of a child of nine or ten years of age, at Douglas, while under the influence of chloroform. The *Sun*, May 12th, reports the death of a woman, aged 68, at the Whitechapel Infirmary whilst under chloroform just as an operation for opening an abscess was about to be performed. The *Eastern Morning News*, May 14th, reports the death at the Hull Royal Infirmary of a man aged 49 whilst under chloroform. He was about to be operated on for varicose veins, but death occurred before the operation was commenced. The *Nottingham Daily Express*, May 19th, reports the death of a child aged two years at the Derby Infirmary whilst chloroform was being administered. The *Manchester Evening News*, May 24th, reports the death under chloroform of a woman, aged 27, at Pendlebury. The *Sussex Daily News*, May 25th, reports the death under chloroform of a man, aged 55, at the Sussex County Hospital. The *Blackburn Telegraph*, June 1st, reports the death of a man aged 57, at the Blackburn Infirmary. He had been taking the chloroform for three or four minutes, when he suddenly ceased breathing before he was under its influence. The *Birmingham Gazette*, June 2nd, reports an inquest on a man aged 54, suffering from strangulated hernia, who died under chloroform and ether.

